# Thyroid hormone and vitamin D regulate VGF expression and promoter activity

**DOI:** 10.1530/JME-15-0224

**Published:** 2016-02

**Authors:** Jo E Lewis, John M Brameld, Phil Hill, Dana Wilson, Perry Barrett, Francis J P Ebling, Preeti H Jethwa

**Affiliations:** 1 Division of Nutritional Sciences, School of Biosciences, University of Nottingham, Sutton Bonington Campus, Loughborough, LE12 5RD, UK; 2 School of Life Sciences, University of Nottingham, Queen's Medical Centre, Nottingham , NG7 2UH, UK; 3 The Rowett Institute of Nutrition and Health, University of Aberdeen, Bucksburn, Aberdeen, AB21 9SB, UK

**Keywords:** VGF (non-acronymic), thyroid hormone, SH-SY5Y cells, Siberian hamster, vitamin D

## Abstract

The Siberian hamster (*Phodopus sungorus*) survives winter by decreasing food intake and catabolizing abdominal fat reserves, resulting in a sustained, profound loss of body weight. Hypothalamic tanycytes are pivotal for this process. In these cells, short-winter photoperiods upregulate deiodinase 3, an enzyme that regulates thyroid hormone availability, and downregulate genes encoding components of retinoic acid (RA) uptake and signaling. The aim of the current studies was to identify mechanisms by which seasonal changes in thyroid hormone and RA signaling from tanycytes might ultimately regulate appetite and energy expenditure. proVGF is one of the most abundant peptides in the mammalian brain, and studies have suggested a role for VGF-derived peptides in the photoperiodic regulation of body weight in the Siberian hamster. *In silico* studies identified possible thyroid and vitamin D response elements in the VGF promoter. Using the human neuroblastoma SH-SY5Y cell line, we demonstrate that RA increases endogenous VGF expression (*P*<0.05) and VGF promoter activity (*P*<0.0001). Similarly, treatment with 1,25-dihydroxyvitamin D_3_ increased endogenous VGF mRNA expression (*P*<0.05) and VGF promoter activity (*P*<0.0001), whereas triiodothyronine (T_3_) decreased both (*P*<0.01 and *P*<0.0001). Finally, intra-hypothalamic administration of T_3_ blocked the short day-induced increase in VGF expression in the dorsomedial posterior arcuate nucleus of Siberian hamsters. Thus, we conclude that VGF expression is a likely target of photoperiod-induced changes in tanycyte-derived signals and is potentially a regulator of seasonal changes in appetite and energy expenditure.

## Introduction

The Siberian hamster (*Phodopus sungorus*) has been increasingly used to investigate hypothalamic mechanisms regulating energy homeostasis due to its natural seasonal cycle of appetite, energy expenditure, and body weight (Ebling 2014). These hamsters naturally become obese in the summer long-day photoperiod (LD), but then enter a catabolic state during winter short-day photoperiod (SD) where they reduce their food intake and catabolize intra-abdominal fat reserves, subsequently losing up to a third of their body weight (Bartness *et al*. 1989, Klingenspor *et al*. 1996, Mercer *et al*. 2001). The mechanism(s) by which these long-term changes in body weight are regulated are poorly understood, but they are clearly distinct from those governing short-term homeostatic regulation of appetite (Ebling 2015). The expression of a number of genes has been shown to be altered in the ventral ependymal wall lining the hypothalamic third ventricle of Siberian hamsters housed in different photoperiods (Barrett *et al*. 2005, 2006). Much recent interest has focused on tanycytes as the key component of the ependyma, as these cells are clearly important nutrient sensors in the hypothalamus, and also a stem cell niche (Bolborea & Dale 2013, Robins *et al*. 2013). For example, SD photoperiod upregulates expression of deiodinase 3 (DIO3), the enzyme responsible for degrading active 3,5,3′-triiodothyronine (T_3_) to inactive 3,3′-diiodothyronine (T_2_), as well as the conversion of thyroxine (T_4_) to the inactive 3,3′,5-triodothyronine, also called reverse T_3_ or rT_3_ (Barrett *et al*. 2005, 2006, 2007). A number of genes encoding components of retinoic acid (RA) uptake and signaling are also downregulated by SD in tanycytes (Ross *et al*. 2004, Shearer *et al*. 2010). The major question now arises as to how seasonal changes in thyroid hormone and RA signaling from tanycytes to hypothalamic neurons ultimately regulate appetite and energy expenditure.

One of the most abundant peptidergic genes expressed in the mammalian brain, and particularly the hypothalamus, is VGF (non-acronymic) (Levi *et al*. 2004, Lewis *et al*. 2015*b*), a gene first identified on the basis of its rapid induction *in vitro* by nerve growth factor (NGF) in PC12 cells, a rat neuroblastic cell line (Levi *et al*. 1985). It has subsequently been shown that RA, in addition to NGF, can act as a transcriptional inducer of the *VGF* gene in SK-N-BE (a human neuroblastoma cell line) and PC12 cells (Salton *et al*. 1991, Rossi *et al*. 1992, Cerchia *et al*. 2006). Hypothalamic VGF mRNA expression is altered by photoperiod in Siberian hamsters, with significantly lower expression in SD compared to LD in the arcuate nucleus (ARC), but intriguingly upregulation in a specific subdivision of the ARC, the dorsomedial posterior ARC (dmpARC), defined by expression of histamine 3 receptors (Barrett *et al*. 2005). After switching to LD, *VGF* expression in the dmpARC decreased rapidly, ahead of body weight changes (Barrett *et al*. 2005). We have previously shown that intracerebroventricular administration of the VGF-derived peptide, TLQP-21, decreased appetite and body weight in Siberian hamsters in LD (Jethwa *et al*. 2007), supporting the view that the products of this gene might impact upon seasonal behavior and physiology. *In silico* analysis of the mouse VGF promoter sequence revealed a potential thyroid response element (TRE), as well as a vitamin D response element (VDRE). We previously demonstrated that thyroid hormone (T_3_) availability in the hypothalamus was likely to be reduced in hamsters in SD, due to increase in DIO3 expression (Barrett *et al*. 2007, Murphy *et al*. 2012), while changes in vitamin D production have also previously been associated with photoperiod, particularly in the human kidney and skin (Webb *et al*. 1988, Holick 1995). Understanding the interactions of these regulatory factors is necessary to establish the mechanisms which promote the catabolic state observed in Siberian hamsters in SD. Thus, we investigated the effects of thyroid hormone (T_3_), RA and vitamin D (1,25-dihydroxyvitamin D_3_ [1,25D_3_]) on VGF mRNA expression and promoter activity *in vitro*. The experimental approach was to use the SH-SY5Y neuroblastoma cell line, a common neuronal cell model due to its ability to differentiate into a more mature neuron-like phenotype and to be propagated unlike primary mammalian neurons (Dwane *et al*. 2013, Kovalevich & Langford 2013). These *in vitro* studies were complemented by an investigation of the effects of intra-hypothalamic implantation of T_3_ on the expression of VGF in the hypothalamus of Siberian hamsters, a procedure previously demonstrated to maintain an anabolic phenotype characteristic of LD exposure (Barrett *et al*. 2007, Murphy *et al*. 2012).

## Methods

### Materials

Unless stated otherwise, all chemicals for cell culture were purchased from Sigma–Aldrich, while those for RNA extraction, complimentary DNA (cDNA) synthesis and quantitative PCR (QPCR) were purchased from Roche Life Science. RA, T_3_, and 1,25D_3_ were obtained from Sigma–Aldrich and NGF was supplied by Millipore (Telecula, CA, USA) and was diluted as per manufacturer's instructions. RA was reconstituted in 95% ethanol at 2.7 mg/ml; subsequent dilutions were made in DMEM with a final ethanol concentration of 0.1% (v/v). NGF (10 μg/ml) was reconstituted in sterile DMEM; subsequent dilutions were made in sterile DMEM. T_3_ was reconstituted in 1.0 ml 1.0 M NaOH (20 μg/ml) and 49 ml sterile DMEM; subsequent dilutions were made in DMEM. 1,25D_3_ was reconstituted in 95% ethanol (10 μM); subsequent dilutions were made in sterile DMEM.

### Cell culture

The human neuroblastoma SH-SY5Y cells (a kind gift from Dr Perry Barrett, but originally from European Collection of Cell Cultures (ECACC) Centre for Applied Microbiology and Research (CAMR), Porton Down Salisbury, Wiltshire, UK) were grown in uncoated 25 cm^2^ tissue culture flasks in DMEM/Ham's F-12 containing 10% fetal bovine serum (FBS), 100 units/l penicillin and 100 mg/l streptomycin (DMEM/F12 complete) maintained at 37  °C in a 95% humidified incubator with 5% CO_2_ (Lewis *et al*. 2015*a*). Although SH-SY5Y cells tend not to adhere very well to uncoated plastic, they were routinely split 1:3 with 0.05% trypsin every 48 h.

### RNA extraction and cDNA synthesis

Prior to harvesting the cells, the DMEM/F12 complete media was removed and cells were harvested in 200 μl of RNase-free PBS. Total RNA was extracted from the cells using the High Pure Isolation Kit (Roche) as described previously (Brown *et al*. 2012). First-strand cDNA was synthesized using the Transcriptor First Strand cDNA Synthesis Kit (Roche), according to the manufacturer's protocol. The cDNA was stored at –20  °C.

### Quantitative RT-PCR

The PCR was performed with SYBR green optimized for the LightCycler 480 (Roche Life Science). All reactions were performed in triplicate on 384 well plates as described previously (Brown *et al*. 2012). Transcript abundance was determined from a standard curve produced using a serial dilution of pooled cDNA made from all samples to check linearity and efficiency of the PCR and the values normalized to cyclophilin A, the most stable reference gene under the experimental conditions. The respective primer sets can be seen in Table 1.

### Synthesis of the VGF promoter and other constructs

First, the ZsGreen gene present in the promoterless pZsGreen1-1 vector (Clontech Laboratory) was replaced with CBG992AmRFP from pCR2CBG992AmRFP (pRFP, Stritzker *et al*. 2014). The subcloning strategy utilized *BamH*I and *Not*I; then both fragments were purified using the QIAquick gel extraction kit (QIAGEN), as per manufacturer's instructions. The pZsGreen1-1 backbone was treated with calf intestinal alkaline phosphatase (Promega) to prevent re-ligation of the plasmid without insert. This new reporter construct was designated pRFP-basic and was transformed into JM109 cells (Promega). Cultures were grown overnight, before plating and colony selection. All purified plasmids were subjected to restriction enzyme digestion and sequencing (performed by Source BioScience, Nottingham, UK) to confirm identity. Subsequently a cytomegalovirus (CMV) promoter, from pLenti6.4-CMV-C/EBPPA (a kind gift from Prof Michael Lomax, University of Nottingham, UK), was cloned into the pRFP-basic vector. Using a similar strategy, pRFP-basic was digested with *Xho*I and *Spe*I, whilst pLenti6.4-CMV-C/EBPPA was digested with *Sal*I (which produces identical overhangs to *Xho*I) and *Spe*I (NEB, Hitchin, Hertfordshire, UK) to obtain the CMV promoter, which was subsequently ligated into the pRFP construct. This new construct was designated pCMV-RFP.

Approximately 1.1 kb of the mouse *VGF* promoter (−1151 bp to +51 bp, relative to the transcriptional start site; accession number: NM_001039385.1) was generated by PCR (see Table 1 for primers). The resultant amplicon was purified using the QIAquick gel extraction kit (QIAGEN), as per manufacturer's instructions, and inserted into the pGEM-T-Easy vector for subsequent subcloning into pRFP-basic using *Spe*I and *Sac*I. Alternatively, *Mbo*I and *Bgl*II were used to create the truncated 0.5 kb promoter construct lacking the potential TRE and VDRE. The orientation and authenticity of these constructs, designated pVGF1.1 and pVGF0.5, were verified by sequencing (performed by Source BioScience, UK).

### Study 1: the long-term maintenance of differentiation of SH-SY5Y cells

The SH-SY5Y cells demonstrate reduced neurite length, a marker of differentiation, when cultured on uncoated surfaces (Dwane *et al*. 2013). Therefore to establish culture conditions for the long-term maintenance of differentiated neuronal cells, SH-SY5Y cells were plated in six-well plates coated with or without 0.01% poly-l-lysine or 10 μg/ml collagen type IV. Cells were plated at 5×10^4^ cells/cm^2^ in DMEM/F12 complete, and 24 h later treated with 10 μM RA in DMEM/F12 complete for 120 h. Differentiation was subsequently maintained by treating the cells with 50 ng/ml NGF (Promega) in DMEM/F12 complete every 48 h. Images were captured 24, 48, 72, 96 and 120 h post-differentiation and neurite length determined using ImagePro Software (version 4.0; Image Pro, Rockville, MD, USA). A differentiated cell was defined as a cell with a neurite length greater than the length of the cell body. At the end of the study, cells were harvested for RNA and subsequent QPCR to determine the expression of the known markers of neurite differentiation, microtubule-associated protein 2 (Map2), Tau and growth-associated protein 43 (GAP43) (Table 1).

### Study 2: regulation of endogenous expression of VGF mRNA *in vitro*

To investigate the regulation of endogenous *VGF* gene expression, undifferentiated SH-SY5Y cells were plated onto uncoated six-well plates for 24 h. Subsequently, DMEM/F12 complete was removed and the cells treated with DMEM/F12 complete containing 50 ng/ml NGF, 10 μM RA, 10 nM T_3_ or 10 nM 1,25D_3_ for 24 h. For studies in differentiated SH-SY5Y cells, plates were coated with 10 μg/ml collagen type IV and differentiated as per study 1. The adherent, differentiated cells were then treated with DMEM/F12 complete containing 50 ng/ml NGF, 10 μM RA, 10 nM T_3_ or 10 nM 1,25D_3_ for 24 h. Cells were harvested for RNA extraction and QPCR to determine endogenous expression of VGF.

### Study 3: regulation of VGF promoter activity *in vitro*

To investigate the regulation of the *VGF* promoter in undifferentiated cells, SH-SY5Y cells were plated onto uncoated six-well plates and grown to 70% confluence, prior to transfection in DMEM/F12 basic medium (DMEM/F12 containing 2.5% FBS, without antibiotics). The undifferentiated cells were transfected with the various plasmids using the FuGENE HD (reagent: DNA ratio of 3:1) as per manufacturer's instructions. Briefly, undifferentiated cells were co-transfected with the VGF promoter construct (pVGF1.1 or pVGF0.5) and tenfold less pZsGreen1-N1 (Clontech Laboratories), a plasmid containing a variant of green fluorescent protein (GFP) under the control of a CMV promoter, with the latter used to correct for differences in transfection efficiency. pCMV-RFP (i.e. a strong promoter) was used as a positive control, while pRFP-basic (a promoterless plasmid) was used as a negative control, with both again being co-transfected with pZsGreen1-N1 to normalize for variability in transfection efficiencies. Seventy-two hours post-transfection, undifferentiated cells were switched back to DMEM/F12 complete (with antibiotics) containing 50 ng/ml NGF, 10 μM RA, 10 nM T_3_, or 10 nM 1,25D_3_ for 48 h. Promoter activities (fluorescence) were quantified at different time points using the Typhoon Trio+ (GE Healthcare, Little Chalfont, Buckinghamshire, UK).

In the experiments using differentiated cells, SH-SY5Y cells were grown to 70% confluence on uncoated six-well plates and transfected as before. The cells were then harvested using 0.05% trypsin, plated onto six-well plates coated with 10 μg/ml type IV collagen for 24 h, before differentiation was induced with 10 μM RA for 120 h (as before). Transfecting cells prior to differentiation have been shown to result in higher transfection efficiencies without altering the course of transgene expression (Lahousse *et al*. 2006, Chu *et al*. 2009). Transfected, differentiated cells were then treated with 50 ng/ml NGF, 10 μM RA, 10 nM T_3_, or 10 nM 1,25D_3_ for 48 h to investigate effects on the VGF promoter activities (up to 120 h post-transfection). Promoter activities (fluorescence) were quantified at different time points using the Typhoon Trio+ (GE Healthcare).

### Study 4: the effects of intra-hypothalamic T_3_ administration on VGF mRNA expression in Siberian hamsters exposed to LD or SD

Hypothalamic expression of VGF was studied in tissues collected in a study previously described by Barrett *et al*. (2007). The study was carried out in age-matched adult male Siberian hamsters obtained from a colony bred in house (Ebling 1994), individually housed at constant temperature (21±1 ^o^C) and 40–50% humidity. Animals had access *ad libitum* to food (Teklad 2019, Harland, UK) and water throughout the studies. All animal procedures were approved by the University of Nottingham Local Ethical Review Committee and were carried out in accordance with the UK Animals (Scientific Procedures) Act of 1986 (Project License PPL 40/2372).

Anesthetized Siberian hamsters (aged 3–4 months) maintained in LD had either T_3_ (mixture of crystalline T_3_ and medical grade silicone/Silastic-brand adhesive) or sham (medical grade silicone/Silastic-brand adhesive alone) microimplants placed bilaterally into the hypothalamus (6.5 mm below the surface of the dura at 0.5 mm to the left of the midline as defined by the center of the superior midsagittal sinus) as previously described (Barrett *et al*. 2007). At 12–16 days post-surgery, the Siberian hamsters were subdivided according to bodyweight to be either maintained in LD (*n*=6/group) or transferred into SD (*n*=7–8/group). Animals were euthanized with sodium pentobarbital (Euthatal: Rhone Merieux, Harlow) at 8 weeks post-surgery, and *in situ* hybridization studies for VGF were carried out as previously described in Barrett *et al*. (2005).

To determine expression slides were scored for the density of silver grains over individual cells in the dmpARC reflecting hybridization of the VGF probe by an observer who was blind to the treatment: 0=no hybridization, 1=a few cells expressing VGF mRNA, 2=moderate VGF mRNA expression cells, 3=abundant VGF mRNA. Scores were not possible for three animals as sections containing the dmpARC region were not available.

### Statistical analysis

Data represent the means±s.e.m. of six technical replicates (i.e. wells). Significant differences between groups for dependent variables were tested using either an unpaired, two-tailed Student's *t*-test, one-way ANOVA in studies 1 and 2 or a two-way ANOVA (treatmentxtime) in study 3. In study 4, scores were analyzed by a Kruskal–Wallis test with *post hoc* Dunn's tests for multiple comparisons. Changes in body weight (data represent the means±s.e.m.) and in paired testis weights over the course of the T_3_ treatment were compared using one-way ANOVA with *post hoc* Dunnett's tests for multiple comparisons. Statistical analyses were conducted using GraphPad PRISM (version 6.0, San Diego, CA, USA). Significance was accepted at *P*<0.05.

## Results

### Study 1: the long-term maintenance of differentiation of SH-SY5Y cells

**Figure 1:**
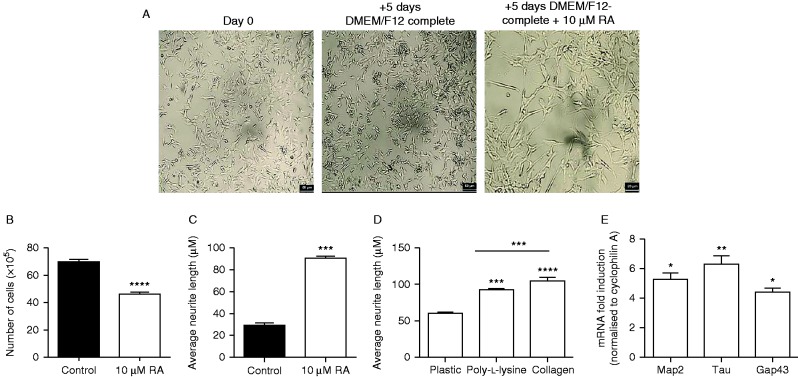
Treatment of the SH-SY5Y cell line with 10 μM RA reduces cell proliferation and increases differentiation. (A) Images of SH-SY5Y cells at days 0 and 5 of differentiation in the absence or presence of 10 μM RA, showing differences in cell numbers and morphology (neurite lengths). (B) Treatment of the SH-SY5Y cell line with 10 μM RA significantly decreased cell number (*P*<0.0001) and (C) significantly increased neurite length (a marker of differentiation) (*P*<0.0001). (D) Neurite length was significantly greater 5 days post-treatment with differentiation media in wells coated with poly-l-lysine or type IV collagen (*P*<0.0001) than plastic. (E) Treatment of the SH-SY5Y cell line with differentiation media significantly increased Map2, Tau and Gap43 (neuronal markers of differentiation). Gene expression was quantified by QPCR, normalized to cyclophilin A mRNA, and then compared to the normalized expression in undifferentiated cells. All values are means±s.e.m. (*n*=6, *P<0.05, **P<0.01, ***P<0.001, and **** *P*<0.0001).

Treatment of the SH-SY5Y cells with 10 μM RA for 5 days significantly reduced proliferation (Fig. 1A and B, *P*<0.01). The cells underwent a significant change in morphology, with the length of neurites significantly increasing in response to 10 μM RA (Fig. 1A and C, *P*<0.0001). By coating the cell culture wells with an extracellular matrix protein such as poly-l-lysine or type IV collagen, neurite outgrowth was significantly enhanced (Fig. 1D, *P*<0.0001). Expression of Map2, Tau, and Gap43 mRNA were all significantly increased in comparison to undifferentiated controls (Fig. 1E, *P*<0.05). To ensure a homogenous population of differentiated SH-SY5Y cells, differentiated cells were then treated with 50 ng/ml NGF every 48 h. Under these conditions, cultures of differentiated cells could be maintained for up to 20 days without reversion or cell death.

### Study 2: regulation of endogenous expression of VGF mRNA *in vitro*

**Figure 2:**
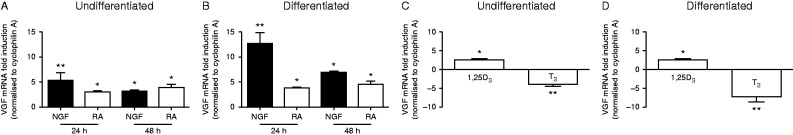
Treatment of undifferentiated or differentiated SH-SY5Y cells with NGF, RA or 1,25D_3_ increases endogenous VGF mRNA expression, whereas T_3_ decreases endogenous VGF mRNA. VGF mRNA was significantly increased by treatment with 50 ng/ml NGF or 10 μM RA for 24 and 48 h in both (A) undifferentiated SH-SY5Y cells and (B) differentiated SH-SY5Y cells. VGF mRNA was significantly increased by treatment with 10 nM 1,25D_3_ but significantly reduced with 10 nM T_3_ in both (C) undifferentiated SH-SY5Y cells and (D) differentiated SH-SY5Y cells. All values are means±s.e.m. (*n*=6, *P<0.05, **P<0.01 for comparisons between control and treatment).

Treatment of undifferentiated SH-SY5Y cells with 50 ng/ml NGF for 24 and 48 h significantly increased VGF mRNA five- and threefold (Fig. 2A, *P*<0.01 and *P*<0.05 vs vehicle treated controls), while treatment with 10 μM RA significantly increased VGF mRNA two- and fourfold at 24 and 48 h, respectively (Fig. 2A, *P*<0.05 vs vehicle treated controls). Similar effects of NGF and RA were observed in differentiated SH-SY5Y cells, but the magnitude of the responses to NGF were bigger (Fig. 2B). Further studies showed that treatment of undifferentiated SH-SY5Y cells with 10 nM 1,25D_3_ for 24 h resulted in a threefold increase in VGF mRNA (*P*<0.05), whereas treatment with 10 nM T_3_ resulted in a fourfold decrease (*P*<0.01) in VGF mRNA (Fig. 2C), with similar effects again observed in differentiated SH-SY5Y cells, although the inhibitory effect of T_3_ tended to be greater (Fig. 2D).

### Study 3: regulation of VGF promoter activity *in vitro*

**Figure 3:**
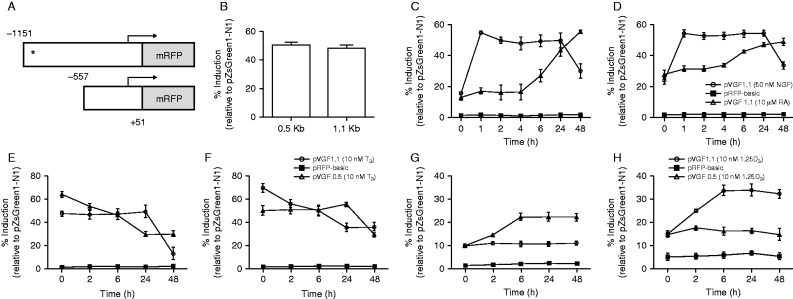
Treatment of undifferentiated or differentiated SH-SY5Y cells with NGF, RA or 1,25D_3_ increases VGF promoter activity, whereas T_3_ reduces VGF promoter activity. (A) The VGF promoter (1.1 kb) was cloned into a mammalian expression vector, based on the backbone of pZsGreen1-1 (Clontech), in which the GFP reporter gene was substituted for an mRFP. A subsequent truncated promoter construct (0.5 kb), which lacked the potential TRE and VDRE (indicated as *), was generated via 5′ deletion. (B) Promoter activities (fluorescence) were similar in cells transfected with either the 1.1 or 0.5 kb VGF promoter constructs. Promoter activities are shown relative to the positive control (pZsGreen1-N1, fluorescence set at 100%). VGF promoter activity (pVGF1.1 construct only) was increased by 50 ng/ml NGF and 10 μM RA in both (C) undifferentiated and (D) differentiated SH-SY5Y cells. (C) SH-SY5Y cells were transfected with pVGF1.1 and treated with NGF or RA 72 h post-transfection. 50 ng/ml NGF rapidly induced pVGF1.1 promoter activity within 1 h (*P*<0.0001). Ten micromolar RA resulted in a slower, yet significant, increase in pVGF1.1 promoter activity, starting 6 h post-treatment (*P*<0.001). (D) Undifferentiated cells were transfected as described in (B), but 72 h post-transfection, cells were differentiated with 10 μM RA for 5 days. Differentiated transfected cells were then treated with 50 ng/ml NGF or 10 μM RA for 48 h. Treatment of differentiated SH-SY5Y cells with NGF (*P*<0.0001) and RA (*P*< 0.0001) had a similar effect to that observed in transfected undifferentiated cells. VGF promoter activity (pVGF1.1 construct only) was decreased by 10 nM T_3_ in both (E) undifferentiated and (F) differentiated SH-SY5Y cells, but there were no effects on the pVGF0.5 promoter construct (which lacked the potential TRE). Cells were transfected and differentiated as described for (B) and (D) respectively, but cells were pre-treated with DMEM/F12 complete containing 50 ng/ml NGF for 1 h (to induce promoter activity) prior to addition of 10 nM T_3_, which significantly reduced pVGF1.1 promoter activity (*P*<0.001) in both (E) undifferentiated and (F) differentiated cells. Similarly, treatment with 10 nM 1,25D_3_ significantly increased pVGF1.1 promoter activities (*P*<0.0001) in both (G) undifferentiated and (H) differentiated cells, but there were no effects on the pVGF0.5 promoter construct (which lacked the potential VDRE). pRFP-basic was included as a negative control in all experiments to indicate background fluorescence, as this construct lacks a functional promoter. All values are means±s.e.m. (*n*=6).

Transfection of undifferentiated SH-SY5Y cells with pVGF0.5 or pVGF1.1 (containing 0.5 and 1.1 kb of the 5′ flanking region of the VGF promoter respectively (Fig. 3A)) resulted in significant increases in fluorescence, indicating promoter activity, but there was no difference between them (*P*>0.05, Fig. 3B). Treatment of transfected undifferentiated SH-SY5Y cells with either 10 μM RA or 50 ng/ml NGF resulted in significant increases in VGF promoter activities, with NGF inducing a much faster response than RA (time vs treatment interaction: *F*=27.94, *P*<0.0001, Fig. 3C). Similar time-dependent effects of both NGF and RA were observed in differentiated SH-SY5Y cells (time vs treatment interaction: *F*=30.91, *P*<0.0001, Fig. 3D). In both undifferentiated and differentiated SH-SY5Y cells, treatment with NGF resulted in a rapid induction of VGF promoter activity, whereas RA showed a much slower response with a delayed onset. Given the effect of T_3_ on endogenous VGF mRNA, transfected cells for promoter studies were pre-treated with 50 ng/ml NGF for 1 h (to briefly induce promoter activity), before removal and treatment with 10 nM T_3_. Treatment of undifferentiated or differentiated SH-SY5Y cells with 10 nM T_3_ significantly decreased promoter activity for the pVGF1.1 construct (time vs treatment interaction: *F*=86.13, *P*<0.0001, Fig. 3E and F), while treatment with 10 nM 1,25D_3_ resulted in a significant increase in pVGF1.1 promoter activity (time vs treatment interaction: *F*=14.58, *P*<0.0001, Fig. 3G and H). There were no effects (*P*>0.05) of either 10 nM T_3_ or 10 nM 1,25D_3_ on activity of the truncated plasmid (pVGF0.5), confirming that the response elements for the two nuclear receptors were only present in the longer promoter construct.

### Study 4: the effects of intra-hypothalamic T_3_ administration on VGF mRNA expression in Siberian hamsters exposed to LD or SD

**Figure 4:**
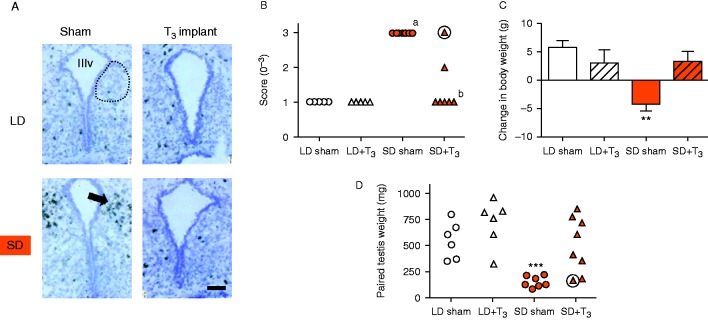
Intra-hypothalamic T_3_ administration reduces VGF mRNA expression in the SD Siberian hamster. (A) representative photomicrographs of coronal sections through the dmpARC counterstained with cresyl violet, *VGF* hybridization is revealed by dark silver grains in the overlying emulsion in Siberian hamsters exposed to LD or SD receiving intra-hypothalamic sham or T_3_ implants for 8 weeks. Dotted line indicates approximate boundaries of the dmpARC, arrow indicates induces expression in a SD sham hamster, scale bar=100 μm. (B) analysis of *VGF* mRNA abundance, scores for individual animals are depicted; ^a^*P*<0.001 vs LD sham group, ^b^*P*<0.05 vs SD sham group. (C) overall change in body weight, values are mean±s.e.m., **P<0.01 vs LD-sham group. (D) individual paired testis weights at the end of the study, ***P<0.001 vs LD-sham group. Weekly mean body weight data and group mean testis weight data have been published previously (Barrett *et al*. 2007). Circled values (panels (B) and (D)) are data from the same individual.

As previously reported (Barrett *et al*. 2007) exposure to SD for 8 weeks induced significant body weight loss (*P*<0.01, Fig. 4C) and testicular regression (*P*<0.001, Fig. 4D), and both these physiological responses to SD were completely blocked by intra-hypothalamic T_3_ implants (Fig. 4C and D). As expected, a very low level of VGF expression was observed in the dmpARC in hamsters which were maintained in LD and received either intrahypothalamic sham or T_3_ implants, that is a very small number of cells in this region expressed VGF mRNA (Fig. 4A and B). In contrast, VGF mRNA expression was abundant in the dmpARC of sham-implanted hamsters exposed to SD for 8 weeks (*P*<0.001, Fig. 4A and B). Intra-hypothalamic implantation of T_3_ significantly blocked this SD-induced increase in VGF mRNA abundance (*P*<0.05, Fig. 4A and B). Intrahypothalamic implantation of T_3_ had no effect on VGF mRNA abundance or on any physiological parameters in hamsters that were maintained in LD (Fig. 4A, B, C, and D). In the one hamster where the SD-induced increase in VGF abundance was not prevented by the T_3_ treatment, the SD-induced involution of the testes was not blocked (Fig. 4D), and the hamster lost 3.3 g of body weight thus the SD-induced catabolic response was not prevented.

## Discussion

Tissue-specific expression of the *VGF* gene has been previously described (Canu *et al*. 1997), but what other regulatory elements are present within the promoter remain to be established (Levi *et al*. 2004). Using the human neuroblastoma SH-SY5Y cell line, we have demonstrated that RA and NGF increase both endogenous *VGF* mRNA expression and VGF promoter activity. The increase confirms the rise in VGF mRNA demonstrated in response to treatment with NGF in PC12 cells and to RA in SK-N-BE cells (Levi *et al*. 1985, Cerchia *et al*. 2006). Whilst the increase in VGF mRNA and promoter activity in response to treatment with NGF was rapid, it was rather transient, decreasing after 24 h. In contrast, treatment with RA resulted in a much slower-, longer-term induction of VGF promoter activity, which continued to increase through to 48 h.

*In silico* studies identified possible TRE and VDRE sequences in the VGF promoter, and our studies have shown that both endogenous VGF mRNA expression and VGF promoter activity are suppressed by T_3_ treatment, but increased with 1,25D_3_. Correspondingly, an *in vivo* study revealed that intra-hypothalamic T_3_ administration via slow-release microimplants reduced VGF mRNA expression in the dmpARC of SD-exposed Siberian hamsters. This suggests that VGF expression *in vivo* may be regulated by availability of these hormones/ligands, which in turn are determined by the transport of their precursors and the enzymes responsible for synthesizing or degrading their active forms. Substantial evidence indicates that the generation of thyroid hormone and RA signals in the mediobasal hypothalamus is regulated by season and photoperiod (discussed by Ebling (2014, 2015)). For example, expression of the genes encoding many of the components of the RA-signaling pathway (cellular retinol-binding protein-1 (CRBP-1), cellular RA-binding protein-2 (CRABP-2) and the nuclear receptors, RAR and RXR) is reduced in response to SD in the Siberian hamster (Ross *et al*. 2005, Barrett *et al*. 2006), so this may explain why VGF mRNA expression is reduced in the hypothalamus of Siberian hamsters exposed to SD (Barrett *et al*. 2005). Furthermore, in a photoperiod-responsive strain of rat, expression of RALDH-1, which converts retinol to RA, is also reduced in the hypothalamus in SD (Shearer *et al*. 2010). Transthyretin (TTR) is a transporter for vitamin A and its metabolite RA as well as T_4_. TTR binds T_4_ to establish a pool of T_4_ in the plasma and cerebral spinal fluid (Prendergast *et al*. 2002) as well as transporting retinol by binding to the RBP (Hyung *et al*. 2010). However, studies utilizing TTR null mice have shown that while there are reductions in retinol and RBP in these mice, they display no symptoms of vitamin A deficiency, suggesting that TTR is not crucial for retinol delivery (Episkopou *et al*. 1993, van Bennekum *et al*. 2001). Thus, local availability of RA is determined by RALDH-1 and components of its signalling pathways (CRBP-1, CRABP-2 and RAR and RXR). All of which, as explained above, are reduced in response to SD in the Siberian hamster (Ross *et al*. 2005, Barrett *et al*. 2006, Shearer *et al*. 2010). Indeed, TTR expression in the hypothalamus of Siberian hamsters has been reported and the responsiveness of the gene was limited to the photorefractory state (Prendergast *et al*. 2002). TTR mRNA is strongly expressed in the ependymal layer of the third ventricle and is decreased in SD (relative to LD) (Helfer *et al*. 2012), thus resulting in reductions in local T_4_ availability. This in combination with the reduction in mRNA expression of DIO2 and increased expression and activity of DIO3 is observed in Siberian hamsters maintained in SD (Watanabe *et al*. 2004, Barrett *et al*. 2007, Herwig *et al*. 2009), and this results in a decrease in T_3_. However, in the absence of RA, we suggest VGF expression would remain low in the hypothalamus of hamsters maintained in SD; thus we propose reduced RA in the hypothalamus of Siberian hamsters in response to SD results in reduced VGF expression (as demonstrated by Barrett *et al*. (2005)).

Previously, both vitamin A and RXR ligands have been shown to influence appetite (Anzano *et al*. 1979, Ogilvie *et al*. 2004). Therefore, the reduction in the availability of RA and components of its signaling pathway in the hypothalamus of Siberian hamsters in SD and subsequent reduction in VGF expression in the hypothalamus is a possible explanation for the effects on appetite. The complexity of the hormonal regulation of VGF expression that the current study has revealed may also explain why in SD there is a local upregulation of VGF expression in the dmpARC. In the hamster, this region has a much higher level of expression of thyroid hormone receptor b1 than the surrounding hypothalamus (Barrett *et al*. 2007), and our current study demonstrates that VGF mRNA expression in the dmpARC is specifically regulated by thyroid hormone. Thus, the SD-induced increase in DIO3 expression in tanycytes would be expected to reduce local T_3_ availability, resulting in a loss of repression of VGF gene expression in the dmpARC. Previously we demonstrated effects of increasing hypothalamic T_3_ availability on the behavior and physiology of SD-exposed Siberian hamsters. Intra-hypothalamic T_3_ implants placed in hamsters in SD produced a rapid increase in body weight, a reflection of increased food intake and a decrease in energy expenditure (Murphy *et al*. 2012). Here, we demonstrate that locally increasing hypothalamic T_3_ blocks the SD-induced increase in *VGF* mRNA expression in Siberian hamsters. This is associated with a blockade of the SD-induced decrease in appetite and in weight loss, and also with the SD-induced inactivation of the reproductive axis. The correlation between *VGF* expression in the dmpARC and the physiological responses to SD was particularly highlighted in one individual hamster where the intrahypothalamic T_3_ implants were ineffective in preventing any of the SD responses, probably because their placement was too rostral to influence hypothalamic function. The question now arises as to the specific role of T_3_-regulated *VGF* expression in driving these seasonal responses. The function of the dmpARC itself is not clear; as one recent study found that SD-induced weight loss could occur in hamsters with lesions of this structure (Teubner *et al*. 2015). However, other lines of evidence suggest that increased VGF expression in the dmpARC could contribute to the SD-catabolic state, for example, at least one of the peptide products (TLQP-21) has been shown to reduce appetite when infused centrally into the hamster (Jethwa *et al*. 2007).

Repression of VGF promoter activity was nullified via the removal of the potential TRE from the promoter construct. TREs have previously been shown to be responsible for the dose-dependent T_3_ repression of *Mc4r* promoter activity (Decherf *et al*. 2010). Furthermore, TREs have been shown to function in combination with RAR/RXR (De Luca 1991). Heterodimerization of the TR with RXR favors the dissociation of suppressors and the recruitment of activators of transcription (Cheung *et al*. 2009). Therefore, we hypothesize that the inability of TR to heterodimerize with RXR results in the repression of VGF in the hypothalamus of SD Siberian hamsters.

Additionally, we demonstrate that 1,25D_3_, the active metabolite of vitamin D, significantly increases VGF endogenous mRNA expression and promoter activity in both undifferentiated and differentiated SH-SY5Y cells. However, further studies are required to determine the effects of 1,25D_3_ *in vivo*. The seasonal regulation of the harderian gland of Siberian hamsters has been shown to be regulated by vitamin D (Perez-Delgado *et al*. 1993, Stumpf *et al*. 1993), while we have shown that plasma vitamin D_3_ levels in adult hamsters are significantly higher in SD than in LD (SI Anderson, M Smith & FJP Ebling, unpublished observations). Interestingly, 1,25D_3_ has been shown to have neuroprotective qualities *in vitro* (Brewer *et al*. 2001, Oermann *et al*. 2004, Wang *et al*. 2004), while treatment of the SH-SY5Y cell line with 1,25D_3_ inhibits proliferation (Celli *et al*. 1999), similar to the effects of RA. However, long-term incubation with 1,25D_3_ only resulted in a slight trend towards differentiation (Celli *et al*. 1999). More recently, Agholme *et al*. (2010) demonstrated that pre-treatment with RA followed by extracellular matrix gel adhesion, in combination with brain-derived neurotrophic factor (BDNF), neuregulin B1, NGF and 1,25D_3_, resulted in differentiated SH-SY5Y cells with unambiguous resemblance to adult neurons. The results presented here support the idea of differentiation-induced expression of the VGF gene and, therefore a possible role in neurogenesis. This agrees with previous studies from Esposito *et al*. (2008), who suggests that receptor tyrosine kinase (RET) activation is a critical step in differentiation. Indeed, Korecka *et al*. (2013) have shown that RA induces RET expression in SH-SY5Y cells, while Cerchia *et al*. (2006) have suggested that inhibition of RET in SK-N-BE cells increases VGF expression. Moreover, a recent study in mice has demonstrated that the VGF-derived peptide, TLQP-62, directly increased the generation of neural progenitor cells in the hippocampus and potentiated BDNF-TrkB signalling (Thakker-Varia *et al*. 2014), thus the concept that enhanced VGF expression promotes neurogenesis and plasticity is rapidly gaining ground.

**Figure 5:**
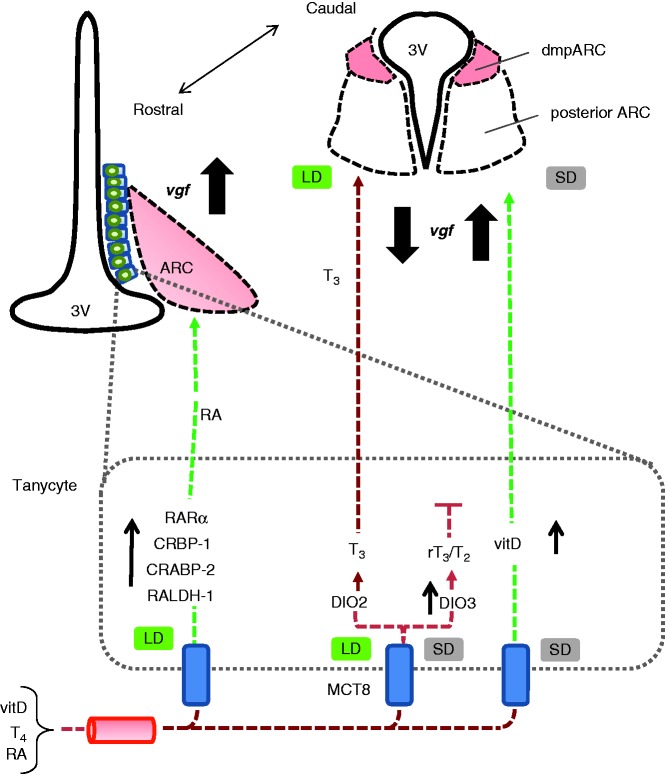
Schematic summary of the proposed photoneuroendocrine control of VGF expression. Thyroxine (T_4_) is taken up from the circulation into tanycytes via MCT8 transporters, and in LD is converted by DIO2 to T_3_ (Ebling 2014). Furthermore, in LD, components of the retinoic acid-signaling pathway – CRBP-1, CRABP-2, RAR, and RXR – are all upregulated in the Siberian hamster (Ross *et al*. 2005, Barrett *et al*. 2006), while RALDH-1 is increased in the photoperiodic rat (Shearer *et al*. 2010). We demonstrate that VGF expression and promoter activity in SH-SY5Y cells is increased in response to treatment with RA and vitamin D and reduced in response to treatment with T_3_. Furthermore, VGF mRNA expression is reduced in response to intra-hypothalamic T_3_ administration in the SD-exposed Siberian hamster. In SD, expression of DIO3 is upregulated, and thus, inactive metabolites of T_4_ such as rT_3_ and T_2_ are produced alongside reductions in components of the retinoic acid-signaling pathway, while vitamin D plasma levels are increased. This may account for the increase in *VGF* expression in the dmpARC whilst reducing *VGF* expression in the ARC. Adapted from Ebling (2014).

In conclusion, this study demonstrates that T_3_ and 1,25D_3_, as well as NGF and RA, regulate endogenous VGF expression and promoter activity *in vitro*, while T_3_ regulates VGF mRNA expression *in vivo*, providing a possible mechanism for the seasonal regulation of appetite in the Siberian hamster (Fig. 5), as well as suggesting a possible role for *VGF* in neurogenesis. Furthermore, it substantiates the central role of T_3_ and 1,25D_3_ in neuroendocrine and metabolic signaling.

## Author contribution statement

J E L: conducted the studies, analyzed the data and drafted the manuscript; J M B: guidance during experiments, analysis of data and reviewed draft manuscript; P H: guidance during experiments; D W and P B: *in situ* hybridisation studies and analysis; F J P E: expertise with regards to seasonal models, *in vivo* study, analysis of data and reviewed draft manuscript; P H J: principal investigator, analysis of results, reviewed draft manuscript and final sign off of manuscript.

## Figures and Tables

**Table 1:** The PCR primers used for QPCR analysis of gene expression or to amplify 1.1 kb of the VGF promoter.

**Gene**	**Forward primer (5′–3′)**	**Reverse primer (5′–3′)**
Cyclophilin A	TCCTGCTTTCAAGAATTATTCC	ATTCGAGTTGTCACAGTCAGC
Map2	CATGGGTCACAGGGCACCTATTC	GGTGGAGAAGGAGGCAGATTAGCTG
Tau	GCGGCAGTGTGCATATAGTCTACA	GGAAGGTCAGCTTGTGGGTTTCAA
Gap43	AGTGAGCAGCGAGCAGAA	GTTGCGGCCTATGAGCTT
*VGF*	GACCCTCCTCTCCACCTCTC	ACCGGCTCTTTATGCTCAGA
VGF promoter	AAGGGTGTGGGAGAGTTGG	GAGGGATGGACAGCGGAG
